# A Year-Book of Therapeutics, Pharmacy and Allied Sciences

**Published:** 1872-08

**Authors:** 


*A Year-Book of Therapeutics, Pharmacy and allied Sciences.* Edited by HORATIO C. WOOD, Jr., M. D. New York: WM. WOOD & Co., 1872.

This is a collection of the more important articles which appeared in the numbers of *New Remedies* for the past year, and is of value both as a book of reference and as a record of advances and discoveries which are yearly being made in the medical sciences.

The editor, Dr. Wood, is a gentleman of large and cultivated experience, and the selections, most of which are from the periodical medical literature, are made with care and taste.

